# Computer-Aided Diagnosis of Spinal Tuberculosis From CT Images Based on Deep Learning With Multimodal Feature Fusion

**DOI:** 10.3389/fmicb.2022.823324

**Published:** 2022-02-23

**Authors:** Zhaotong Li, Fengliang Wu, Fengze Hong, Xiaoyan Gai, Wenli Cao, Zeru Zhang, Timin Yang, Jiu Wang, Song Gao, Chao Peng

**Affiliations:** ^1^Institute of Medical Technology, Peking University Health Science Center, Beijing, China; ^2^School of Health Humanities, Peking University, Beijing, China; ^3^Beijing Key Laboratory of Spinal Disease Research, Engineering Research Center of Bone and Joint Precision Medicine, Department of Orthopedics, Peking University Third Hospital, Beijing, China; ^4^Department of Orthopedic, People’s Hospital of Tibet Autonomous Region, Lhasa, China; ^5^Medical College, Tibet University, Lhasa, China; ^6^Department of Respiratory and Critical Care Medicine, Peking University Third Hospital, Beijing, China; ^7^Tuberculosis Department, Beijing Geriatric Hospital, Beijing, China

**Keywords:** computer-aided diagnosis, spinal tuberculosis, computed tomography, feature fusion, deep learning

## Abstract

**Background:**

Spinal tuberculosis (TB) has the highest incidence in remote plateau areas, particularly in Tibet, China, due to inadequate local healthcare services, which not only facilitates the transmission of TB bacteria but also increases the burden on grassroots hospitals. Computer-aided diagnosis (CAD) is urgently required to improve the efficiency of clinical diagnosis of TB using computed tomography (CT) images. However, classical machine learning with handcrafted features generally has low accuracy, and deep learning with self-extracting features relies heavily on the size of medical datasets. Therefore, CAD, which effectively fuses multimodal features, is an alternative solution for spinal TB detection.

**Methods:**

A new deep learning method is proposed that fuses four elaborate image features, specifically three handcrafted features and one convolutional neural network (CNN) feature. Spinal TB CT images were collected from 197 patients with spinal TB, from 2013 to 2020, in the People’s Hospital of Tibet Autonomous Region, China; 3,000 effective lumbar spine CT images were randomly screened to our dataset, from which two sets of 1,500 images each were classified as tuberculosis (positive) and health (negative). In addition, virtual data augmentation is proposed to enlarge the handcrafted features of the TB dataset. Essentially, the proposed multimodal feature fusion CNN consists of four main sections: matching network, backbone (ResNet-18/50, VGG-11/16, DenseNet-121/161), fallen network, and gated information fusion network. Detailed performance analyses were conducted based on the multimodal features, proposed augmentation, model stability, and model-focused heatmap.

**Results:**

Experimental results showed that the proposed model with VGG-11 and virtual data augmentation exhibited optimal performance in terms of accuracy, specificity, sensitivity, and area under curve. In addition, an inverse relationship existed between the model size and test accuracy. The model-focused heatmap also shifted from the irrelevant region to the bone destruction caused by TB.

**Conclusion:**

The proposed augmentation effectively simulated the real data distribution in the feature space. More importantly, all the evaluation metrics and analyses demonstrated that the proposed deep learning model exhibits efficient feature fusion for multimodal features. Our study provides a profound insight into the preliminary auxiliary diagnosis of spinal TB from CT images applicable to the Tibetan area.

## Introduction

Spinal tuberculosis (spinal TB) is secondary to TB of the lung, gastrointestinal tract, or lymphatic tract, and it causes bone TB *via* the blood circulation route (Garg and Somvanshi, 2011; Rasouli et al., 2012; Khanna and Sabharwal, 2019). The insidious onset of spinal TB and the lack of specificity in clinical manifestations can lead to serious symptoms, such as kyphosis, abscess injection, and spinal instability, further causing paraplegia or death (Qian et al., 2018; Vanino et al., 2020). The incidence of TB is significantly higher in underdeveloped plateau regions, particularly in the Tibetan area of China (Du et al., 2017; Zhu et al., 2017); for example, the rate of reported TB cases in the Tibet Autonomous Region was 166.6 per 100,000 in 2017, which was the highest in China. Spinal TB accounts for approximately 2% of pulmonary TB, 15% of extrapulmonary TB, and 50% of bone and joint TB throughout the world (Fuentes Ferrer et al., 2012). Moreover, the CT manifestation of spinal TB is complicated, including typical manifestations (destruction of the vertebral body, collapse of the vertebral space, abscess compression on the spinal cord or nerve roots, etc.) and atypical manifestations (vertebral body osteoid formation, vertebral body disruption in the anterior column, vertebral body endplate worm-like disruption, pus in the paravertebral soft tissue shadow, continuous unilateral bone disruption, and asymmetry between the imaging manifestations and symptoms) (Cremin et al., 1993; Rauf et al., 2015).

Local grassroots hospitals lack experienced specialists and multimodal medical imaging equipment, they have only CT or digital radiography (DR) machines. Therefore, most Tibetan grassroots doctors cannot make expeditious medical decisions. These poor health conditions lead to high rates of misdiagnosis, missed diagnosis, and delays in effective treatment, which result in severe complications that impose serious social burdens on Tibetan herdsmen (Wang et al., 2015). Computer-aided diagnosis (CAD), including classical machine learning (ML) and deep learning (DL), is an effective method for assisting primary care physicians in treating patients with spinal TB; CAD builds mathematical models on computers using fuzzy mathematics, probability statistics, and even artificial intelligence to process patient information and propose diagnostic opinions and treatment plans. To the best of our knowledge, except for a few reports on the simple application of statistical analysis to the clinical diagnosis of spinal TB (Zhang et al., 2019; Liu et al., 2021), there are limited studies on artificial intelligence-aided diagnosis of spinal TB, including diagnostic classification, pathological grading, lesion segmentation, and prognostic analysis.

Radiomics, a typical example of traditional ML, is an automated high-throughput method that extracts a significant amount of quantitative handcrafted features from medical images (Lambin et al., 2012). These handcrafted features are the conversion of digital images into mineable data and the subsequent analyses of these data for decision support (Gillies et al., 2016), such as color, texture, shape, and statistical characteristics, including scale-invariant feature transform (SIFT), speeded-up robust features (SURF), and oriented rotated brief (ORB) (Abdellatef et al., 2020). Currently, although many handcrafted features have been designed for various clinical applications (Moradi et al., 2007; Aerts et al., 2014; Cook et al., 2014; Wang et al., 2014; Li et al., 2018; Tian et al., 2018; Song et al., 2021), classical ML cannot accurately perform ancillary diagnostics of TB owing to its limited accuracy (Goodfellow et al., 2016; Currie et al., 2019). The design of handcrafted features often involves finding the right trade-off between accuracy and computational efficiency based on the subjective understanding of key issues (Nanni et al., 2017); therefore, an inappropriate handcrafted feature typically results in poor generalization ability (Suzuki et al., 2012), which significantly hinders the development of ML diagnostic systems.

Contrastingly, DL based on convolutional neural networks (CNNs) is another medical CAD method that enhances the identification of subtle differences in radiographical characteristics, and it is feasible for integrating multi-omics medical data by harnessing the power of computing (Altaf et al., 2019; Alkhateeb et al., 2021). Unlike traditional ML, the features extracted by DL can be predetermined by a CNN during training, without elaborate design (Anwar et al., 2018). There are various CNN models that are applicable to different medical scenarios, such as common CNN for grading (Yang et al., 2018; Swarnambiga et al., 2019), U-Net for segmentation (Ronneberger et al., 2015; Jackson et al., 2018; Deng et al., 2021), and GAN for the generation of synthetic images (Lei et al., 2019). The technological innovations of CAD show that DL could be a suitable candidate for auxiliary diagnosis in modern healthcare systems. However, a CNN needs the majority of datasets to extract features automatically and requires significant training time to obtain a reliable model (Goodfellow et al., 2016; Currie et al., 2019), both of which are scarce resources in medical practices. Moreover, the lack of interpretability of DL is another important factor that hinders its development in rigorous clinical work.

Therefore, the effective fusion of the multimodal features extracted from both ML and DL is one of the key directions to further improve the performance of CAD when compared with the counterparts of individuals above. This approach has had several successful applications with medical radiological images, such as in the determination of tumor benignity and malignancy (Antropova et al., 2017; Xie et al., 2018; Khosravi et al., 2021), lesion segmentation (Su et al., 2021), survival prediction (Shboul et al., 2019; Guo et al., 2021), detection of COVID-19 from chest CT images (Wang S.-H. et al., 2021), and cancer diagnosis and prognosis (Chen et al., 2020). By contrast, published studies on spinal TB have mainly focused on the clinical manifestations and surgical protocol of spinal tuberculosis (Garg and Somvanshi, 2011; Zhu et al., 2017; Khanna and Sabharwal, 2019). Different feature fusion methods have been developed for different clinical purposes, such as a Bayesian algorithm-based method that can realize the fusion decision of multiple features (Khaleghi et al., 2013), a sparse representation-based method that can obtain the joint sparse representation of multiple features (Lai and Deng, 2018), and a DL-based method that can strengthen the feature learning process of deep neural networks (Zhang et al., 2021). However, most of the aforementioned fused features are different representations under the same modality owing to the difficulty of multimodal fusion, and in cross-modal learning, it is difficult to implement transfer learning between more than two modalities. Conversely, the gated information fusion network (Arevalo et al., 2017; Kim et al., 2018) ensures that each single modality can work independently and transfer knowledge mutually, and it realizes the effective fusion of multimodal information, including histology images and genomic features (Chen et al., 2020). It adopts the Kronecker product of unimodal feature representations to control the expressiveness of each single feature *via* a gated information attention mechanism.

In this study, a multimodal feature fusion CNN is proposed to classify spinal TB CT images obtained from local grassroots hospitals in the Tibetan area. It provides a breakthrough in the application area of spinal TB auxiliary diagnosis, although it simply implements the classification of tuberculosis-health diagnostic results in spinal TB CT images. Specifically, the proposed network fuses three different elaborate features, namely SIFT, SURF, and ORB, with the DL feature that originates from the convolutional output layer of common CNNs. A new augmentation algorithm for handcrafted features that effectively simulates the data distribution in the feature space is proposed as a substitute for the image augmentation method. Additionally, a model was designed and used to effectively integrate these individual features, which included four different sections: matching network for consistency of different feature dimensions, backbone for sparse representation of features, fallen network for dimensional reduction, and fusion network for hybridizing multimodal features by a gated mechanism. We evaluated the hypothesis that the proposed method can effectively distinguish tubercular cases from healthy images by conducting experiments and performing several analyses. For convenience, from here on, “positive” and “negative” represent tuberculosis and health, respectively. Based on initial hypothesis attempts, further research will be conducted on other auxiliary diagnostics to form a complete auxiliary diagnostic process for spinal TB and solve the long-standing problem of spinal tuberculosis in Tibet.

## Materials and Methods

### Data Collection

A multimodal image dataset was obtained from the People’s Hospital of Tibet Autonomous Region, China, and consisted of DR and CT images of 197 patients with spinal TB acquired between 2013 to 2020. They were screened by two physicians based on basic patient information, medical records, and imaging evaluation, all of which were surgically treated as definite spinal tuberculosis pathology according to the corresponding guidelines about the diagnosis of spinal TB (Hoffman et al., 1993; Liu et al., 2021). The inclusion and exclusion criteria for the spinal TB cases were as follows:

Inclusion criteria:

• Diagnosis of spinal tuberculosis was confirmed by puncture biopsy or postoperative pathological examination;
• Preoperative DR and CT examinations were performed;
• Complete case data (e.g., gender, age, medical history, physical examination, imaging, and pathology data);
• Patients who were first examined in primary care hospitals in less developed areas and had CT imaging data were prioritized for inclusion.

Exclusion criteria:

• Cases suspected of having spinal tuberculosis without pathological examination;
• A history of spinal trauma before the diagnosis of spinal tuberculosis;
• Incomplete case information.

Table 1 presents the patients’ gender, age, and lesion segment. Some patients had multiple site infections; therefore, the total number of female and male patients is not equal to the total number of cases of cervical, thoracic, lumbar, and sacral vertebral infections. It can be seen that middle-aged people (30–59 years) were the most infected among all age groups, and the number of men infected with spinal TB was higher than that of women. The patients presented in this table are the ones who bear the heaviest social and family pressures. Furthermore, the lumbar vertebrae are the most susceptible to spinal TB infection; therefore, the current research was mainly conducted on the TB of lumbar vertebrae.

**TABLE 1:** Information of patient with spinal TB.

Age	Gender		Lesion segment			
	Female	Male	Cervix	Thorax	Lumbar	Sacrum
10–19	3	7	0	4	4	3
20–29	12	14	0	9	19	1
30–39	18	22	0	19	23	6
40–49	24	19	1	19	24	3
50–59	18	27	1	29	16	4
60–69	10	13	1	13	9	2
70–79	3	6	0	5	4	0
80–89	0	1	0	0	1	0
Sum	88	109	3	98	100	19

Although X-ray examinations are widely used in various primary hospitals, they provide limited information. CT examinations are approximately 20–25 times more sensitive than X-ray-based tissue density tests and are currently one of the most effective clinical bone examination methods. Spiral electron CT provides a high-resolution visualization of the destruction, hyperplasia, sclerosis, and focal boundaries of vertebral bone. Furthermore, it reveals the position of dead bone, fragmented bone, and their protrusion into the spinal canal, showing paravertebral abscesses and their density. Moreover, the lumbar spine has the highest incidence of tuberculosis as it has the most mobility and bears the heaviest burden along the entire spine, as shown in Table 1. For the initial research of spinal TB, a total of 3,000 CT images of the lumbar vertebrae were randomly selected from the abovementioned multimodal image dataset based on slice level, which included a set of 1,500 slices for negative and positive cases. Finally, a small dataset of spinal TB CT images was obtained to explore the flexibility of CAD on spinal TB CT images.

### Feature Selection

Feature engineering is a key step in the supervised classification of pathology images that directly affects the final classification result. Image feature extraction is the premise of image analysis, which is the most effective way to simplify the expression of high-dimensional image data. Based on the above qualitative diagnostic characteristics from orthopedists, three handcrafted features were extracted from spinal TB CT images, including three types of feature descriptors of the vertebral column in CT slices: SIFT (Lowe, 2004), SURF (Bay et al., 2006), and ORB (Rublee et al., 2011). In addition to the handcrafted features mentioned above, deep features were extracted from the convolutional layers and fully connected layers of CNNs. These elaborate features are required for initial preprocessing to ensure dimensional consistency between different features before extracting the respective image characteristics. To understand the varied descriptions of different features, the meaning of the diverse features is indicated as follows.


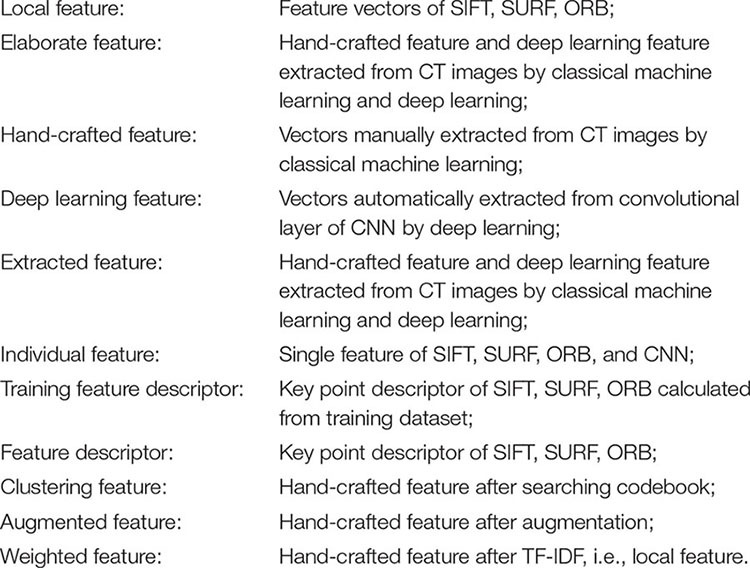


#### Local Features Described With Scale-Invariant Feature Transform, Speeded-Up Robust Features, and Oriented Rotated Brief

Several key points, such as the points of corners and edges, highlights, and dark spots, in an image do not change with luminance, transformation, and noise. These image feature points, which are typically used for image matching and image recognition, can reflect the essential features of an image. Scale-invariant feature transform (SIFT), speeded-up robust features (SURF), and oriented rotated brief (ORB) are three widespread methods used to describe these local feature points. Speckle and corner are just two typical feature points that can reflect key information that exists in the image. Speckle points usually refer to areas with color and grayscale that are different from the surrounding regions. Corner points are the intersection of two edges in the stable and informative areas of an image, which have certain characteristics, such as rotation invariance, scale invariance, affine invariance, and illumination invariance. These feature descriptors have been applied to various medical scenarios, such as medical image classification (Khan et al., 2015), medical image stitching (Singla and Sharma, 2014; Win and Kitjaidure, 2018), medical image fusion (Wang L. et al., 2021), medical image registration (Lukashevich et al., 2011; Li et al., 2012), and medical image retrieval (Govindaraju and Kumar, 2016).

Scale-invariant feature transform uses the Difference of Gaussian (DoG) matrix, which is a speckle detection method, to detect scale-space extrema, and uses an orientation histogram to extract the key point direction. The essence of the SIFT algorithm is to identify the key points and calculate their directions in different scale-spaces. The key points found by SIFT are almost speckle points that cannot be changed by illumination, affine transformation, or noise, such as highlights in dark areas and dark spots in bright areas.

Speeded-up robust features is a scale and rotation invariant descriptor on based on SIFT. Rather than choosing the difference of a Gaussian matrix to detect scale-space extrema in SIFT, it calculates an approximation of the Laplacian of the Gaussian by a Hessian matrix. Instead of using an orientation histogram in SIFT, Harris wavelet response, a corner detection algorithm, is used to assign key point orientations in SURF. Therefore, the key points found by SURF are significantly different from the speckle points found by SIFT. The number of key points detected by SURF is more than that detected by SIFT, whereas the vector dimension (64) of SURF is less than the length (128) of SIFT.

As a very fast binary descriptor based on two algorithms, Features from Accelerated Segment Test (FAST) (Khan et al., 2015) and Binary Robust Independent Elementary Features (BRIEF) (Calonder et al., 2010), ORB is an improved algorithm that outperforms the SIFT and SURF algorithms in terms of nearest-neighbor matching and description efficiency. FAST was used to extract the corner points whose pixel gray value is obviously different from the pixel gray value in the surrounding fields, and BRIEF was employed to describe the points that were extracted by FAST. It has the least number of feature points and the lowest dimensionality (32) of extracted features. In summary, it is a fast feature extracting and matching algorithm with poor quality compared with SIFT and SURF.

**FIGURE 1:**
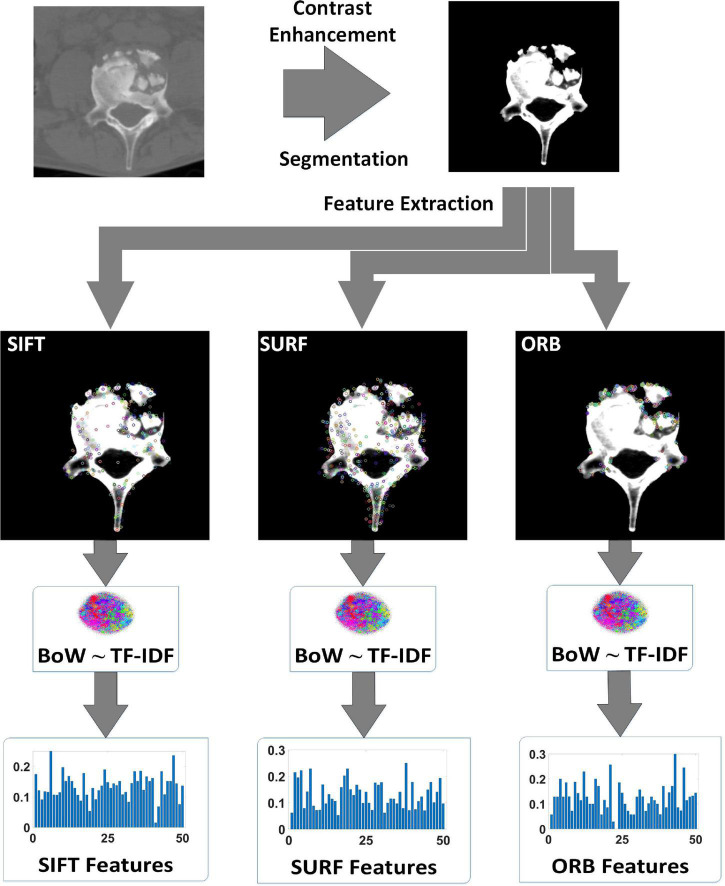
Process of extracting local features of tuberculosis images.

Before extracting feature points, the original spinal TB CT images were enhanced by adjusting the window width and position, and the vertebral region was isolated by watershed segmentation, which had a clear presentation on the centrum and also eliminated noise interference from non-skeletal areas. Subsequently, the feature points of SIFT, SURF, and ORB were transported to the bag of words (BoW) and term frequency–inverse document frequency (TF-IDF) models to obtain fifty-dimension feature vectors, as illustrated in Figure 1. The BoW and TF-IDF models with virtual augmentation are explored in section “Feature Preprocessing.” Finally, we obtained three eigenvectors, which are the local features of all TB images.

#### Deep Learning Features

In addition to the above handcrafted features, the DL characteristics extracted from the convolutional layer of the CNN were another critical feature that contained highly abstract image features. It is generally assumed that there is a closer spatial connection between local pixels than the counterparts between pixels at a greater distance. Thus, each neuron only needs to perceive the local areas of the image and not the global image. Consequently, the global information is obtained by combining the local information at a higher level. A variety of CNNs have been applied to various medical image processing tasks, such as ResNet, VGG, and DenseNet, and thus, the DL features also differ from each other. Because of the black box property of DL features (Guidotti et al., 2018), different CNNs were selected to form the backbone of the proposed network to explore the optimal classification performance of spinal TB CT images.

**FIGURE 2:**
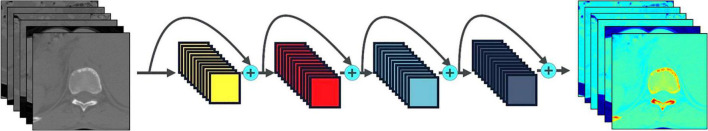
Process of extracting the deep learning features of tuberculosis images. The series of images on the left are the raw CT data, the series of images on the right are the feature maps of the convolution layer of the CNN, and the middle represents the common CNNs.

Figure 2 shows the procedure for extracting DL features. Because the TB image dataset was small, the models that had more or fewer parameters tended to overfit or underfit, respectively; that is, the neural networks with different numbers of layers, ResNet-18 and ResNet-50, VGG-11 and VGG-16, DenseNet-121, and DenseNet-161, were selected as the backbone of the proposed network to avoid overfitting or underfitting.

### Feature Preprocessing

**FIGURE 3:**
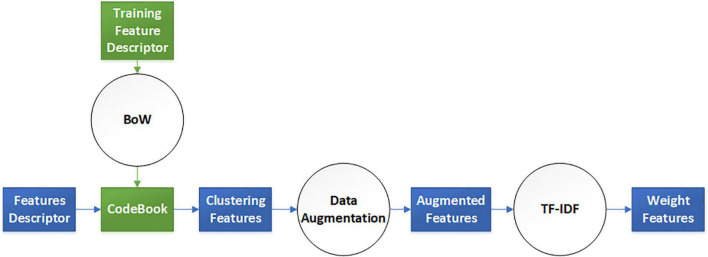
Virtual augmentation model with bag of words (BoW) and term frequency–inverse document frequency (TF-IDF).

The elaborate features should be preprocessed for dimensional consistency between different features. The identical hand-crafted features of each slice were stacked vertically into one larger characteristic set. Subsequently, we used two algorithms, BoW and TF-IDF, to handle the low-dimension characteristic set extracted from the single-scale image. BoW adopted the K-means clustering method for unsupervised clustering of a large number of extracted SIFT, SURF, and ORB key points. The features with strong similarities were classified into the same clustering category. TF-IDF is the product of term frequency (TF) and IDF; it indicates the weight vector of features, where TF is the frequency of occurrence of a feature among all features, and IDF represents the uniqueness of a feature. Figure 3 shows a flowchart illustrating the preprocessing of features. First, we used the key points feature descriptor of SIFT, SURF, and ORB from the training sets to build CodeBook using BoW. The clustering features were the statistics on the number of occurrences of each category after clustering in the feature descriptors by searching the CodeBook. The number of categories was set to 50 after several pretraining experiments with individual features. Second, a new data augmentation algorithm was proposed to improve the generalization of small datasets, and the Algorithm 1 describes the data augmentation methods, which were only applicable to the cluster features processed by the BoW model. Specifically, the clustering information of each feature point was calculated using CodeBook, and the perturbation noise that obeys the normal distribution was used to jitter the clustering information for data augmentation, which increased the generalizability of the dataset. Finally, TF-IDF implemented feature weighting, which counted the frequency information of each feature vector appearing in the augmented feature sets. None of the augmented feature vectors existed in real TB images; that is, only the virtual key points of SIFT, SURF, and ORB existed.

**Table:** 

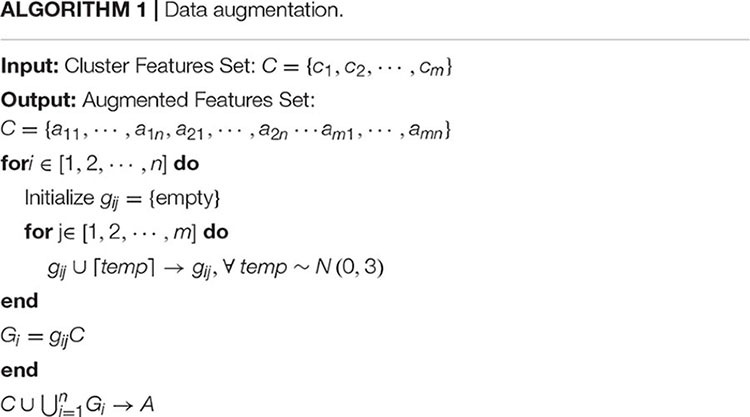

### Fusion Convolutional Neural Network Construction

After comprehensively considering the characteristics of vertebral morphology, we extracted four features from slice images, including the SIFT, SURF, and ORB vectors, and CNN features. Although the extracted handcrafted and DL features cover a wide range of valuable information involving both the local tissue and global slice, an effective method is imperative to fuse these features from different scales to improve prediction accuracy such that it is superior to the corresponding figures of any single feature.

**FIGURE 4:**
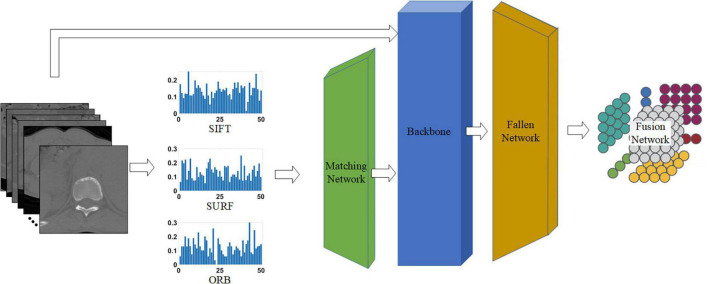
Proposed network for classifying tuberculosis images. The middle three histograms are the SIFT, SURF, ORB vectors with length of 50 extracted from raw CT images, and the green, blue, and orange block represent the matching network, backbone, and fallen network, respectively. The matching network and fallen network are illustrated in Figures 5A,B, respectively, and the last block is the fusion network, which is illustrated in Figure 5C.

As shown in Figure 4, the proposed network consists of four phases: the matching network that adjusts handcrafted features, backbone (i.e., different common CNNs) for processing all features, fallen network for dimension reduction, and fusion network for blending different characteristics. Each network is explained in the following sections.

#### Matching Network

Inconsistencies were present in the characteristic dimensions between the handcrafted and the DL features. Specifically, all handcrafted features were stacked into one-dimensional features with a size of 1 × 50, which was inconsistent with the dimensions of the DL features. Therefore, a matching network was required to reconcile the contradictions in the feature sizes between handcrafted and DL features, that is, to convert one-dimensional vectors into two-dimensional ones.

**FIGURE 5:**
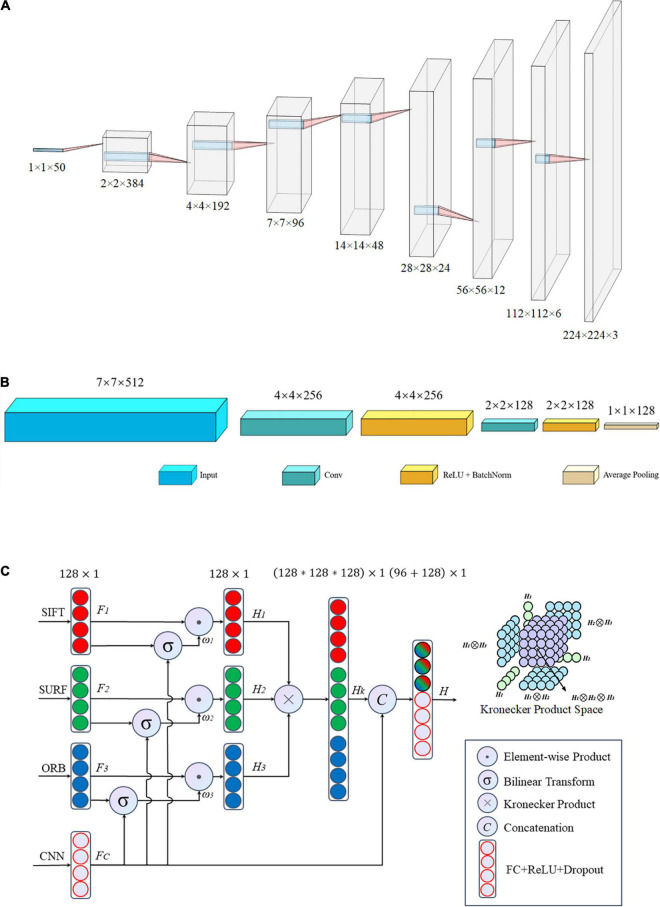
Architecture of the proposed network: **(A)** matching network, **(B)** fallen network, and **(C)** gated information fusion network. The upper right corner of panel **(C)** is the Kronecher space of interactions among three features *H*_1_,*H*_2_, and *H*_3_.

The matching network consisted of nine convolutional blocks, with each block including a fractionally strided convolution, batch normalization, and ReLU activation function. The detailed architecture of the matching network is shown in Figure 5A. The one-dimensional feature with size 1 × 1 × 50 was mapped to a two-dimensional vector of size 224 × 224 × 3, which is similar to the common input size of CNN architectures such as ResNets and to the two-dimensional space of the DL features. Hence, it is easier to tune hyperparameters and fuse handcrafted and DL features.

#### Fallen Network

After the matching network, the handcrafted image features were transformed into the same dimensional space as that of the DL features. Subsequently, a common network was employed to hybridize these different vectors including handcrafted and DL features. This integrated network includes two foundational networks: a backbone network and fallen network. Various CNNs, such as ResNet (He et al., 2016), DenseNet (Huang et al., 2017), and VGG-Net (Simonyan and Zisserman, 2015), serve as the backbone, and they have exhibited outstanding performance in different applications. The fallen network includes two convolutional operations and one average pooling, as shown in Figure 5B. It was used to refine the output characteristics of the backbone by mapping the outputs into a low-dimensional space, that is, a two-dimensional space with a size of 7 × 7 fell into a one-dimensional space with a size of 1 × 1 in detail. Essentially, we obtained a series of one-dimensional feature vectors for the subsequent processing of the fusion network.

#### Gated Information Fusion Network

All image features, including the handcrafted and DL features, were eventually converted into one-dimensional vectors of length 128 after the fallen network was processed. There was high collinearity between the handcrafted and DL characteristics; therefore, an early fusion method that gates the weight contribution of the different tensors at the feature level was used to blend the aforementioned four image features before making a pathological diagnostic evaluation for the final classification.

The structure of the gated fusion network is shown in Figure 5C. For each feature tensor from SIFT, SURF, ORB, and CNN, the dimensions of the input vectors *F*_1_, *F*_2_, *F*_3_, and *F*_C_, respectively, are gradually reduced through the fully connected layer network with a dropout rate of 0.5. For the same dimension, because of the connection between individual captured features, the feature expressions of each handcrafted tensor are weighted by the gated mechanism *via* a combination with DL features to reduce the size of the feature space. The gated mechanism consists of two pathways: one is a one-dimensional vector *F*_i_ with a size of 128 × 1 after the ReLU activation function, and the other vector ω_*i*_ of length 128 is the output of the bilinear transform between *F*_i_ and CNN features *F*_C_, which evaluates the importance of each feature *F*_i_ relative to the more precise CNN features by this non-linear correlation. Subsequently, the Kronecher product, which models the interaction of different features across modalities, constructs a threefold Cartesian space defined by *H*_1_,*H*_2_, and *H*_3_, that is, SIFT, SURF, and ORB, respectively. It also captures the trimodal interactions of all possible unimodal combinations, as shown in the upper right corner of Figure 5C. Finally, the predicted vectors, with a size of 96, and *F*_C_, with a size of 128, are vertically stacked into a larger one-dimensional vector with a length of 224. Subsequently, the predicted values of classification for the TB images are obtained after the fully connected layer operating on the former concatenated one-dimensional vector.

The detailed operations above are summarized as shown below.


(1) $$\omega_{i}=\sigma \,\left(F_{i},F_{C}\right),i=1,2,3$$

(2) $$H_{i}=\text{RELU}\,\left(f\,\left(F_{i}\,⊙\omega_{i}\right)\right),i=1,2,3$$

(3) $$H_{m}=H_{1}\,⊗H_{2}\,⊗H_{3}$$

(4) $$H_{k}=\text{RELU}\,\left(f\,\left(H_{1}\,⊗H_{2}\,⊗H_{3}\right)\right)$$

(5) $$H_{C}=\text{RELU}\,\left(f\,\left(\left[H_{k},F_{C}\right]\right)\right)$$

where, [*x*,*y*] denotes the concatenation of *x* and *y*, and


(6) σ⁢(x,y)=x⁢A⁢y+b

(7) $$f\,\left(x,y\right)=x\,A^{T}+b$$

## Results

### Convergence of the Proposed Model With Different Backbones

A total of 3,000 spinal TB CT images were obtained and subsequently divided into two 1,500 datasets of positive and negative CT slices. For each type of CT image, 900, 300, and 300 slices were randomly selected as the training, validation, and test sets from the small TB dataset, respectively. In terms of training parameters, the optimizer was stochastic gradient descent (SGD) with a momentum of 0.9 and weight decay of 0.001, the learning rate was set to 0.01, which decayed by 0.1 every 7 epochs, and the loss function was a cross-entropy loss function describing the distance between two probability distributions. In addition, three different common deep CNNs (DCNNs) were used as the backbone: ResNet, VGG, and DenseNet. For each DCNN, two main networks with different numbers of layers were used to train on our small TB dataset to generate different sizes of models: 18 vs. 50 layers for ResNet, 11 vs. 16 layers for VGG, and 121 vs. 161 layers for DenseNet. The running environment was Pytorch 1.8.0, CUDA 11.1, and Python 3.7.1 based on Windows 10 with an advanced hardware configuration in terms of the GPU and CPU, i.e., GeForce RTX 3090 and Intel Xeon W-2255, respectively.

**FIGURE 6:**
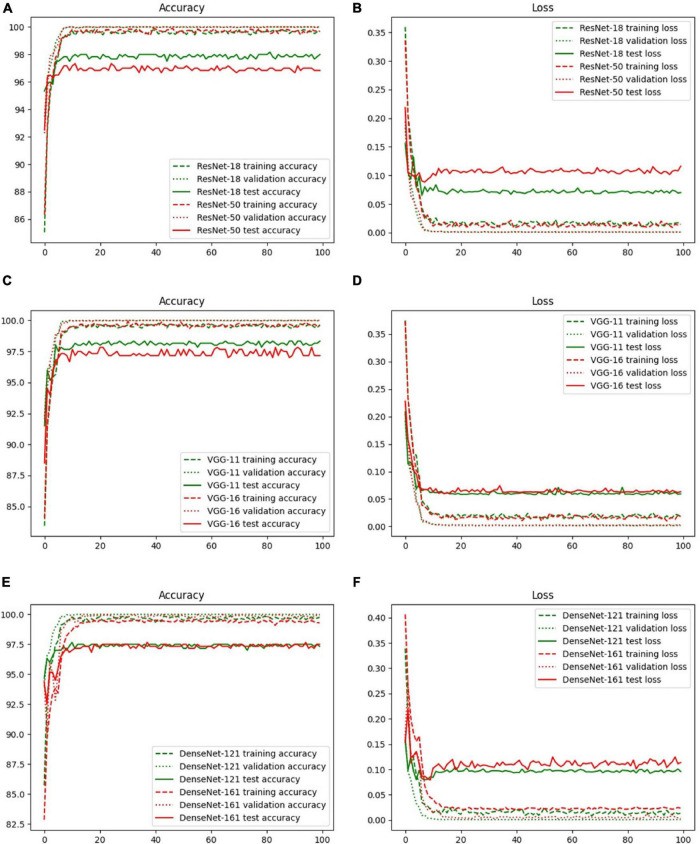
Accuracy and loss curves with different backbones: **(A)** ResNet-18, **(B)** ResNet-50, **(C)** VGG-11, **(D)** VGG-16, **(E)** DenseNet-121, and **(F)** DenseNet-161.

The accuracy and loss curves of the training, validation, and test sets are shown in Figure 6. The accuracy curves clearly appear to level off, and the loss curves converge to equilibrium with slight fluctuations starting at epoch 10. Specifically, the test loss curve of ResNet-50 is above the ResNet-18 loss curve, which indicates that the corresponding accuracy curve has a lower position compared with that of ResNet-18. Although there are few differences between the loss curves of VGG-11 and VGG-16, the accuracy is the same as that of ResNet, that is, the more layers in the network, the lower is the test accuracy value. However, there was a slight difference between DenseNet-121 and 161. These phenomena are explained in the Discussion, and the evaluation indicators of these three backbones, including accuracy, specificity, sensitivity, and area under the curve (AUC), are discussed in the next section.

### Performance of the Proposed Network With Different Backbones

There are four different quantitative indicators, namely accuracy, specificity, sensitivity, and AUC, that illustrate the predictive performance on 600 test images, as shown in Table 2. The variates of accuracy, specificity, and sensitivity reflect the proportion of all samples with correct predictions, all negative samples with correct predictions, and all positive samples with correct predictions for all actual samples, all actual positive samples, and all actual negative samples, respectively. The AUC is the area enclosed by the coordinate axis under the receiver operating characteristic (ROC) curve. The proposed model with the backbone VGG exhibited the best performance compared with the other models, particularly VGG-11 achieved an accuracy of 98.33%, specificity of 98.33%, sensitivity of 98.33%, and AUC of 99.84%. Although the proposed model with ResNet-50 had the worst accuracy compared with the other models, the AUC was higher than that of ResNet-18, which demonstrated the existence of a superior classification threshold value for ResNet-18. For DenseNet, there were few significant differences between DenseNet-121 and DenseNet-161, both of which had an acceptable performance with an accuracy of 97.67%. Specifically, DenseNet-121 effectively predicted positive samples, whereas DenseNet-161 accurately predicted negative samples. This is due to the higher sensitivity of the former and the higher specificity of the latter.

**TABLE 2:** Performance of the proposed network with different backbones.

Backbone	Accuracy	Specificity	Sensitivity	AUC
ResNet-18	0.9817	0.9800	**0.9833**	0.9960
ResNet-50	0.9733	0.9700	0.9767	0.9967
VGG-11	**0.9833**	0.9833	**0.9833**	**0.9984**
VGG-16	0.9783	0.9833	0.9783	0.9983
DenseNet-121	0.9767	0.9767	0.9767	0.9976
DenseNet-161	0.9767	**0.9867**	0.9667	0.9971

*The bold figures represent the maximum value of each evaluation index.*

**FIGURE 7:**
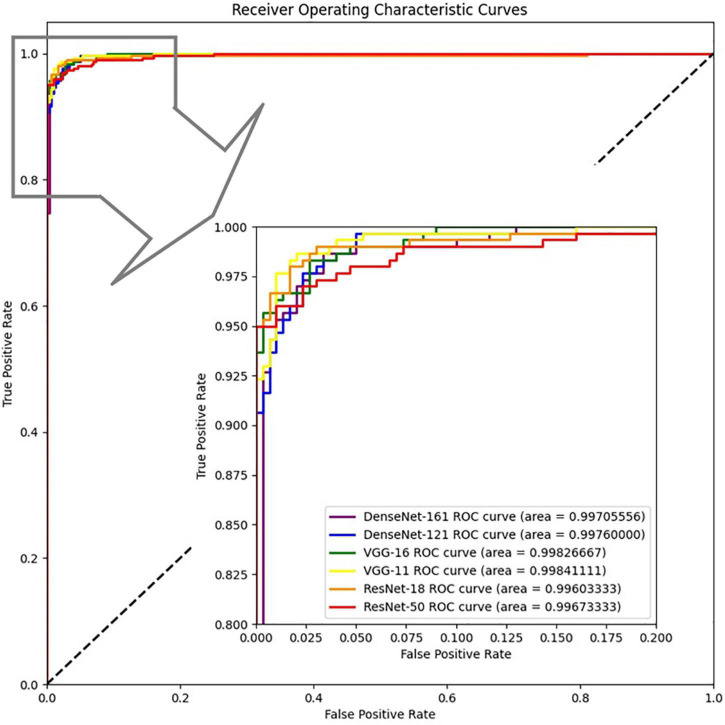
ROCs of the proposed network with different backbones: ResNet-18, ResNet-50, VGG-11, VGG-16, DenseNet-121, and DenseNet-161.

**FIGURE 8:**
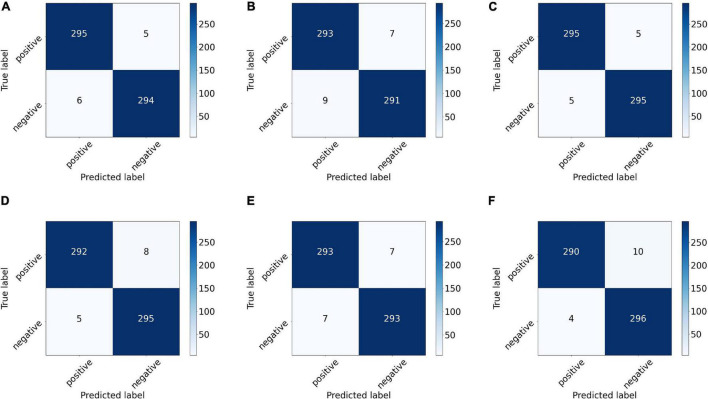
Confusion matrix of the proposed network with different backbones: **(A)** ResNet-18, **(B)** ResNet-50, **(C)** VGG-11, **(D)** VGG-16, **(E)** DenseNet-121, and **(F)** DenseNet-161. The number on each sub-block represents the number of predictions, and the bars on the right of each block represent the heat value chart of the predicted numbers.

We also drew the ROC curves and calculated the AUC of the proposed model with different backbones, as shown in Figure 7 in which the above quantitative indices (including specificity, sensitivity, and AUC) are visualized as the false positive rate, true positive rate, and AUCs. It provides a more intuitive comparison of the differences among these networks when focusing on the upper left area. VGG11 (yellow line) was closest to the perfect classification point in the upper left corner, where the anticipated true positive rate and false positive rate were under different classified threshold values. More importantly, we provide the confusion matrix for each network in Figure 8, in which we can observe the number of correct identifications and the number of incorrect identifications for each category in detail. There was a total of 600 CT images, including 300 positive samples and 300 negative samples. 295 true positives (TP) for VGG-11 and ResNet-18 and 296 true negative (TN) for DenseNet-161 were the maximum of all correctly classified sample volumes, which represents the recognition capability for health and disease. Similarly, the five false positives (FP) for VGG-11 and ResNet-18 and four false negatives (FN) for DenseNet-161 were the minimum of all incorrectly classified sample volumes. Overall, VGG-11 had the highest TP and comparatively higher TN and relatively lower FN, and there was a balanced capacity in predicting negative and positive samples, which demonstrated that VGG-11 was the optimal selection.

Overall, we recommend VGG-11 as the backbone of the proposed deep network for the auxiliary diagnosis of TB CT images based on accuracy, stability, and convergence of the loss function among the six backbones. The subsequent section discusses the analysis conducted on VGG-11.

### Data Augmentation for Handcrafted Features

**FIGURE 9:**
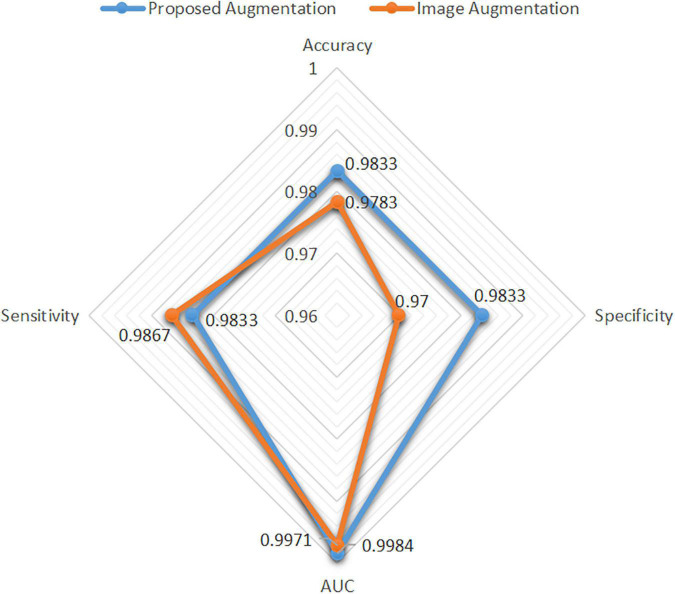
Performance difference between the proposed augmentation and image augmentation.

The image augmentation was similar to the real data distribution in the feature space. In this study, a new data augmentation method was proposed to simulate a real data distribution. The performance of spinal TB classification with the proposed augmentation and image augmentation is shown in Figure 9, highlighting the strength of the proposed augmentation algorithm. The accuracy, specificity, and AUC of the proposed augmentation were all slightly higher than those of image augmentation, and the sensitivity of the former was slightly lower than that of the latter. Generally, the radar map of image augmentation was surrounded by the proposed augmentation; therefore, the proposed method showed significant superiority over direct augmentation on images.

## Discussion

### Model Size vs. Accuracy

**FIGURE 10:**
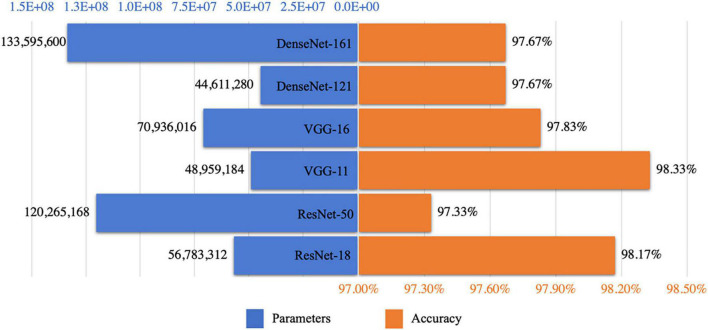
Correlation between model size and training accuracy.

We employed ResNet, VGG, and DenseNet as the backbones. The model layers influenced the classification accuracy, as shown in Figure 6. Figure 10 shows a strong correlation between the number of parameters owned in the selected DCNN models and the prediction accuracy of test sets, which caused underfitting or overfitting when the DL model was too simple or complex to make accurate predictions for unrelated features from the small dataset. For ResNet and VGG, a decline was observed with the increase in parameters, as shown in Figure 10, demonstrating that the excessive number of network layers in DCNN leads to model overfitting. By contrast, the model size had no impact on the accuracy of DenseNet, and the short paths from the initial layers to subsequent layers of DenseNet alleviated the vanishing gradient problem, which ensured maximum information transmission between layers in the network. Essentially, VGG exhibited optimal training performance. In particular, VGG-11 had a superior test accuracy of 98.33% compared with others.

### Individual Features vs. Fusion Feature

**FIGURE 11:**
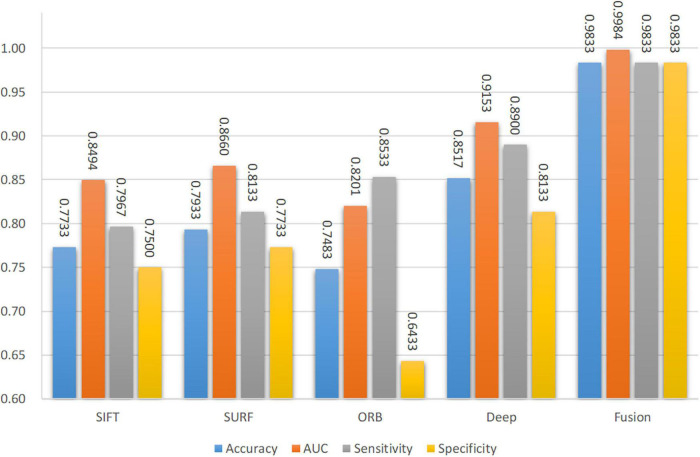
Performance of single features and fusion features.

The four main characteristics were extracted from the CT images to identify spinal TB, namely three handcrafted features and one DCNN feature, i.e., SIFT, SURF, ORB of the local features, and deep features. As illustrated in Table 2, accurate classification performances were obtained by fusing the four different features based on different backbones, particularly for VGG-11. A thorough investigation was conducted to show the significant influence of individual features on the proposed network. As a comparison of the fusion feature, we analyzed the performance of each feature separately based on the proposed network with backbone VGG-11 in Figure 11. Diverse performances were obtained from various characteristics. A common trait was that not all handcrafted features outperformed the deep feature. Furthermore, the four evaluation indicators, namely accuracy, sensitivity, specificity, and AUC, were significantly different in one individual; however, none of them exceeded 90%. This shortcoming was effectively addressed when these different handcrafted features and deep features were fused by the proposed DCNN with the backbone VGG-11, as depicted in the last block of Figure 11. Specifically, the accuracy, AUC, sensitivity, and specificity of deep features improved from 85.17%, 91.53%, 89.00%, and 81.33–98.33%, 99.84%, 99.33%, and 98.33%, respectively, with assistance from the other three handcrafted features.

### Real Image Augmentation vs. Virtual Data Augmentation

**FIGURE 12:**
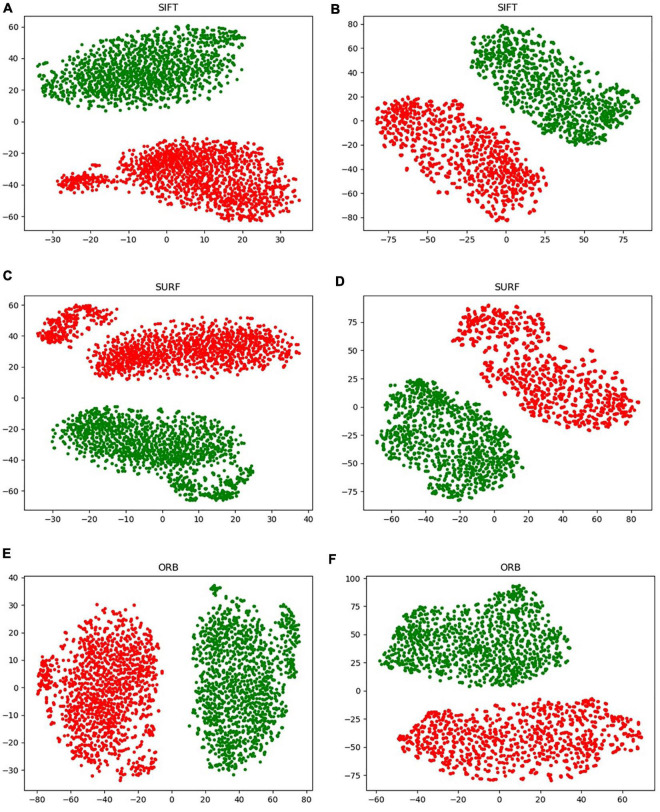
Visualization of the handcrafted features through T-SNE. **(A,C,E)** Virtual data augmentation of SIFT, SURF, and ORB, respectively; **(B,D,F)** real image augmentation of SIFT, SURF, and ORB, respectively.

A new data augmentation method for handcrafted features was proposed based on the algorithm, as described in section “Data Augmentation for Handcrafted Features.” The direct augmentation of images is a common method of data amplification and can produce an augmented feature dataset after extracting the handcrafted features from augmented images. Moreover, it has an identical data scale as the proposed augmentation algorithm. Figure 9 illustrates an intuitive comparison of these two augmentation schemes using a radar map from the four indices. In this study, we conducted a visual analysis of the retained original information in a low-dimensional feature space through t-distributed stochastic neighbor embedding (T-SNE), as shown in Figure 12. In column b, that is, the T-SNE visualization of image augmentation, there are irregular gaps within the same category and considerable overlap among neighboring data points. This demonstrates that the CT slices obtained from image augmentation do not fully represent the real data distribution. By contrast, the binary data distribution (i.e., the red and green points) of the proposed augmentation (column a) is more uniform than that of image augmentation (column b), except for several outliers. This proves that the newly generated feature points can effectively fill the missing data in the spatial distribution.

### Comparison of Heatmap Between Common Convolutional Neural Networks and the Proposed Network

**FIGURE 13:**
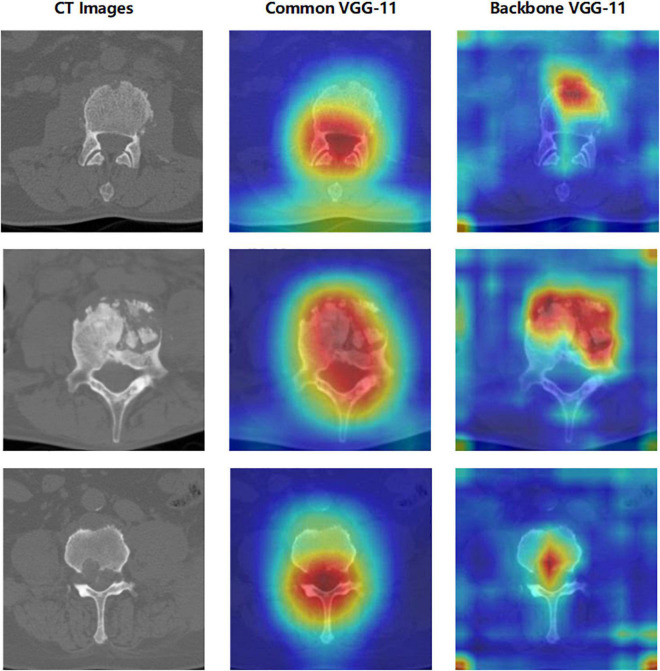
Heatmap of the convolution layer weight visualization based on Grad-CAM++. The left column contains the original CT images, the middle column contains the heatmap generated by common VGG-11, and the right column contains the heatmap generated by backbone VGG-11 of the proposed network.

In Figure 11, a significant improvement can be observed when the CNN features from VGG-11 fused three different handcrafted features. The accuracy increased from 85.17% to 98.33%. Compared with the direct classification of VGG-11 on CT images, some changes were observed in the region of interest for the proposed fusion model with VGG-11 as the backbone. To explore the differences in the area of interest between these two models, Grad-CAM++ (Chattopadhay et al., 2018) was used to generate a heatmap++, as shown in Figure 13. Significant differences can be observed between these two methods on the heatmap of model concerns. VGG-11 focused on the vertebral foramen region in the TB images, regardless of negative or positive cases, which created a significant distraction for the classified judgment. By contrast, the proposed fusion model focused on the areas of destruction of vertebral bodies, even though some unrelated regions received little attention from the fusion models, which had less of an adverse effect on the final classification.

## Conclusion

This study proposes a novel DL-based classification model by fusing four image features, including three handcrafted features and one CNN feature—SIFT, SURF, ORB, and the CNN feature. During the feature engineering phase, the BoW and TF-IDF algorithms combined with a new data augmentation algorithm were used to extract the three handcrafted features, and the deep features were extracted from the convolution layers of common DCNNs, including ResNet, VGG, and DenseNet. The proposed network consists of four main sections: matching network, backbone, fallen network, and fusion network. Specifically, the matching network is used to adjust the dimensions of handcrafted features to match the image size, the fallen network integrates and processes each single feature from two-dimensional into one-dimensional vectors, and the fusion network is composed of a gated information fusion network and Kronecher space, which realizes the effective fusion of different characteristics and outputs the final classification results of TB images. Experimental results were obtained using different backbones: ResNet-18/50, VGG-11/16, and DenseNet-121/201. The results demonstrated that VGG-11 achieved the optimal performance in terms of accuracy, AUC, specificity, and sensitivity. Furthermore, we analyzed the performance of the individual features, the proposed augmentation algorithm, the model stability, and the model-focused heatmap to prove the advancement of the proposed network. The proposed method is interpretable in multimodal feature fusion and can be extended to more medical scenarios, which may aid clinical radiologists, particularly grassroots physicians. It has promising potential, although our research was limited to the positive and negative classification of spinal TB CT images. In subsequent studies, the patient clinical data, including gender, age, and medical history, have a strong relationship for the classification of spinal TB, it is worth adding this personal feature into the fusion networks. In addition, we aim to extend the proposed method to CT images that include all types of spines, such as thoracic, sacral, cervical, and lumbar vertebrae. Further exploration will be conducted for DR images based on spinal TB CT images, which can form a more complete auxiliary diagnosis system applicable to grassroots hospitals in Tibet, China.

## Data Availability Statement

The original contributions presented in the study are included in the article/supplementary material, further inquiries can be directed to the corresponding author/s.

## Ethics Statement

The studies involving human participants were reviewed and approved by Ethics Committee of People’s Hospital of Tibet Autonomous Region, China. The patients/participants provided their written informed consent to participate in this study.

## Author Contributions

ZL: conceptualization, data curation, software, and writing original draft preparation. FW: conceptualization, data acquisition, and funding acquisition. FH and ZZ: data acquisition. XG, WC, TY, and JW: conceptualization and manuscript revision. SG: conceptualization, supervision, manuscript review and funding acquisition. CP: data acquisition and funding acquisition. All authors contributed to the article and approved the submitted version.

## Conflict of Interest

The authors declare that the research was conducted in the absence of any commercial or financial relationships that could be construed as a potential conflict of interest.

## Publisher’s Note

All claims expressed in this article are solely those of the authors and do not necessarily represent those of their affiliated organizations, or those of the publisher, the editors and the reviewers. Any product that may be evaluated in this article, or claim that may be made by its manufacturer, is not guaranteed or endorsed by the publisher.

## References

[B1] Abdellatef E. Omran E. M. Soliman R. F. Ismail N. A. Abdelrahman S. E. S. E. Ismail K. N. (2020). Fusion of deep-learned and hand-crafted features for cancelable recognition systems. *Soft. Comput.* 24 15189–15208. 10.1007/s00500-020-04856-1

[B2] Aerts H. J. W. L. Velazquez E. R. Leijenaar R. T. H. Parmar C. Grossmann P. Carvalho S. (2014). Decoding tumour phenotype by noninvasive imaging using a quantitative radiomics approach. *Nat. Commun.* 5:4006. 10.1038/ncomms5006 24892406 PMC4059926

[B3] Alkhateeb A. Tabl A. A. Rueda L. (2021). “Deep learning in multi-omics data integration in cancer diagnostic,” in *Deep Learning for Biomedical Data Analysis*, ed. Elloumi M. (Cham: Springer), 255–271.

[B4] Altaf F. Islam S. M. S. Akhtar N. Janjua N. K. (2019). Going deep in medical image analysis: concepts, methods, challenges, and future directions. *IEEE Access* 7 99540–99572. 10.1109/access.2019.2929365

[B5] Antropova N. Huynh B. Q. Giger M. L. (2017). A deep feature fusion methodology for breast cancer diagnosis demonstrated on three imaging modality datasets. *Med. Phys.* 44 5162–5171. 10.1002/mp.12453 28681390 PMC5646225

[B6] Anwar S. M. Majid M. Qayyum A. Awais M. Alnowami M. Khan M. K. (2018). Medical Image Analysis using Convolutional Neural Networks: a Review. *J. Med. Syst.* 42:226. 10.1007/s10916-018-1088-1 30298337

[B7] Arevalo J. Solorio T. Montes-y-Gómez M. González F. A. (2017). Gated multimodal units for information fusion. *arXiv* [preprint]. Available Online at: https://arxiv.org/abs/1702.01992 (accessed September, 2021).

[B8] Bay H. Tuytelaars T. Van Gool L. (2006). “SURF: speeded up robust features,” in *Computer Vision – ECCV 2006*, eds Leonardis A. Bischof H. Pinz A. (Berlin Heidelberg: Springer), 404–417.

[B9] Calonder M. Lepetit V. Strecha C. Fua P. (2010). “BRIEF: binary robust independent elementary features,” in *Proceedings of the 11th European conference on Computer vision: part IV*, (Heraklion, Crete, Greece: Springer-Verlag).

[B10] Chattopadhay A. Sarkar A. Howlader P. Balasubramanian V. N. (2018). “Grad-CAM++: generalized Gradient-Based Visual Explanations for Deep Convolutional Networks,” in *2018 IEEE Winter Conference on Applications of Computer Vision (WACV)*, (Lake Tahoe, NV, USA: IEEE), 839–847.

[B11] Chen R. J. Lu M. Y. Wang J. Williamson D. F. K. Rodig S. J. Lindeman N. I. (2020). Pathomic Fusion: an Integrated Framework for Fusing Histopathology and Genomic Features for Cancer Diagnosis and Prognosis. *IEEE Trans. Med. Imaging* 1. 10.1109/TMI.2020.3021387 32881682 PMC10339462

[B12] Cook G. J. R. Siddique M. Taylor B. P. Yip C. Chicklore S. Goh V. (2014). Radiomics in PET: principles and applications. *Clin. Transl. Imaging* 2 269–276. 10.1007/s40336-014-0064-0

[B13] Cremin B. J. Jamieson D. H. Hoffman E. B. (1993). CT and MR in the management of advanced spinal tuberculosis. *Pediatr. Radiol.* 23 298–300. 10.1007/BF02010920 8414759

[B14] Currie G. Hawk K. E. Rohren E. Vial A. Klein R. (2019). Machine learning and deep learning in medical imaging: intelligent imaging. *J. Med. Imaging Radiat. Sci.* 50 477–487. 10.1016/j.jmir.2019.09.005 31601480

[B15] Deng Y. Wang C. Hui Y. Li Q. Li J. Luo S. (2021). CTSpine1K: a large-scale dataset for spinal vertebrae segmentation in computed tomography. *arXiv* [preprint]. Available Online at: https://arxiv.org/abs/2105.14711v3 (accessed September, 2021).

[B16] Du H. Cai G. Ge S. Ci W. Zhou L. (2017). Secondary laryngeal tuberculosis in Tibet China: a report of six cases. *Otolaryngol. Case Rep.* 2 26–28. 10.1016/j.xocr.2017.02.004

[B17] Fuentes Ferrer M. Gutiérrez Torres L. Ayala Ramírez O. Rumayor Zarzuelo M. del Prado González N. (2012). Tuberculosis of the spine. A systematic review of case series. *Int. Orthop.* 36 221–231. 10.1007/s00264-011-1414-4 22116392 PMC3282843

[B18] Garg R. K. Somvanshi D. S. (2011). Spinal tuberculosis: a review. *J. Spinal Cord Med.* 34 440–454. 10.1179/2045772311y.0000000023 22118251 PMC3184481

[B19] Gillies R. J. Kinahan P. E. Hricak H. (2016). Radiomics: images are more than pictures, they are data. *Radiology* 278 563–577. 10.1148/radiol.2015151169 26579733 PMC4734157

[B20] Goodfellow I. Bengio Y. Courville A. (2016). *Deep Learning.* Cambridge, Massachusetts: MIT Press, 1–20.

[B21] Govindaraju S. Kumar G. P. R. (2016). A novel content based medical image retrieval using SURF features. *Indian J. Sci. Technol.* 9 1–8. 10.17485/ijst/2016 33041601

[B22] Guidotti R. Monreale A. Ruggieri S. Turini F. Giannotti F. Pedreschi D. (2018). A survey of methods for explaining black box models. *ACM Comput. Surv.* 51:93. 10.1145/3236009

[B23] Guo W. Liang W. Deng Q. Zou X. (2021). A multimodal affinity fusion network for predicting the survival of breast cancer patients. *Front. Genet.* 12:709027. 10.3389/fgene.2021.709027 34490038 PMC8417828

[B24] He K. Zhang X. Ren S. Sun J. (2016). “Deep residual learning for image recognition,” in *2016 IEEE Conference on Computer Vision and Pattern Recognition*, (Las Vegas, NV, USA: IEEE).

[B25] Hoffman E. B. Crosier J. H. Cremin B. J. (1993). Imaging in children with spinal tuberculosis. A comparison of radiography, computed tomography and magnetic resonance imaging. *J. Bone Joint Surg. Br.* 75 233–239. 10.1302/0301-620x.75b2.8444943 8444943

[B26] Huang G. Liu Z. Van Der Maaten L. Weinberger K. Q. (2017). “Densely connected convolutional networks,” in *Proceedings of the IEEE Conference on Computer Vision and Pattern Recognition (CVPR)*, (Honolulu, HI, USA: IEEE), 4700–4708.

[B27] Jackson P. Hardcastle N. Dawe N. Kron T. Hofman M. S. Hicks R. J. (2018). Deep learning renal segmentation for fully automated radiation dose estimation in unsealed source therapy. *Front. Oncol.* 8:215. 10.3389/fonc.2018.00215 29963496 PMC6010550

[B28] Khaleghi B. Khamis A. Karray F. O. Razavi S. N. (2013). Multisensor data fusion: a review of the state-of-the-art. *Inf. Fusion* 14 28–44. 10.1016/j.inffus.2011.08.001

[B29] Khan S. Yong S. Deng J. D. (2015). “Ensemble classification with modified SIFT descriptor for medical image modality,” in *2015 International Conference on Image and Vision Computing New Zealand (IVCNZ)*, (Auckland, New Zealand: IEEE), 1–6.

[B30] Khanna K. Sabharwal S. (2019). Spinal tuberculosis: a comprehensive review for the modern spine surgeon. *Spine J.* 19 1858–1870. 10.1016/j.spinee.2019.05.002 31102727

[B31] Khosravi P. Lysandrou M. Eljalby M. Li Q. Kazemi E. Zisimopoulos P. (2021). A deep learning approach to diagnostic classification of prostate cancer using pathology–radiology fusion. *J. Magn. Reson. Imaging* 54 462–471. 10.1002/jmri.27599 33719168 PMC8360022

[B32] Kim J. Koh J. Kim Y. Choi J. Hwang Y. Choi J. (2018). “Robust deep multi-modal learning based on gated information fusion network,” in *Asian Conference on Computer Vision*, eds Jawahar C. Li H. Mori G. Schindler K. (Cham: Springer), 90–106.

[B33] Lai Z. Deng H. (2018). Medical image classification based on deep features extracted by deep model and statistic feature fusion with multilayer perceptron. *Comput. Intell. Neurosci.* 2018:2061516. 10.1155/2018/2061516 30298088 PMC6157177

[B34] Lambin P. Rios-Velazquez E. Leijenaar R. Carvalho S. Van Stiphout R. G. P. M. Granton P. (2012). Radiomics: extracting more information from medical images using advanced feature analysis. *Eur. J. Cancer* 48 441–446. 10.1016/j.ejca.2011.11.036 22257792 PMC4533986

[B35] Lei Y. Harms J. Wang T. Liu Y. Shu H. K. Jani A. B. (2019). MRI-only based synthetic CT generation using dense cycle consistent generative adversarial networks. *Med. Phys.* 46 3565–3581. 10.1002/mp.13617 31112304 PMC6692192

[B36] Li S. Jiang H. Wang Z. Zhang G. Yao Y.-D. (2018). An effective computer aided diagnosis model for pancreas cancer on PET/CT images. *Comput. Methods Prog. Biomed.* 165 205–214. 10.1016/j.cmpb.2018.09.001 30337075

[B37] Li Z. Kurihara T. Matsuzaki K. Irie T. (2012). “Evaluation of medical image registration by using 3D SIFT and phase-only correlation,” in *international MICCAI Workshop on Computational and Clinical Challenges in Abdominal Imaging*, (Heidelberg: Springer), 255–264.

[B38] Liu X. Zheng M. Sun J. Cui X. (2021). A diagnostic model for differentiating tuberculous spondylitis from pyogenic spondylitis on computed tomography images. *Eur. Radiol.* 31 7626–7636. 10.1007/s00330-021-07812-1 33768287

[B39] Lowe D. (2004). Distinctive image features from scale-invariant keypoints. *Int. J. Comput. Vis.* 60 91–110.

[B40] Lukashevich P. V. Zalesky B. A. Ablameyko S. V. (2011). Medical image registration based on SURF detector. *Pattern Recognit. Image Anal.* 21:519. 10.1134/S1054661811020696

[B41] Moradi M. Mousavi P. Abolmaesumi P. (2007). Computer-aided diagnosis of prostate cancer with emphasis on ultrasound-based approaches: a review. *Ultrasound Med. Biol.* 33 1010–1028. 10.1016/j.ultrasmedbio.2007.01.008 17482752

[B42] Nanni L. Ghidoni S. Brahnam S. (2017). Handcrafted vs. non-handcrafted features for computer vision classification. *Pattern Recognit.* 71 158–172. 10.1016/j.patcog.2017.05.025

[B43] Qian X. Nguyen D. T. Lyu J. Albers A. E. Bi X. Graviss E. A. (2018). Risk factors for extrapulmonary dissemination of tuberculosis and associated mortality during treatment for extrapulmonary tuberculosis. *Emerg. Microbes Infect.* 7:102. 10.1038/s41426-018-0106-1 29872046 PMC5988830

[B44] Rasouli M. R. Mirkoohi M. Vaccaro A. R. Yarandi K. K. Rahimi-Movaghar V. (2012). Spinal tuberculosis: diagnosis and management. *Asian Spine J.* 6 294–308. 10.4184/asj.2012.6.4.294 23275816 PMC3530707

[B45] Rauf F. Chaudhry U. R. Atif M. ur Rahaman M. (2015). Spinal tuberculosis: our experience and a review of imaging methods. *Neuroradiol. J.* 28 498–503. 10.1177/1971400915609874 26450101 PMC4757228

[B46] Ronneberger O. Fischer P. Brox T. (2015). “U-Net: convolutional networks for biomedical image segmentation,” in *Medical Image Computing and Computer-Assisted Intervention – MICCAI 2015*, eds Navab N. Hornegger J. Wells W. M. Frangi A. F. (Cham: Springer), 234–241.

[B47] Rublee E. Rabaud V. Konolige K. Bradski G. (2011). “ORB: an efficient alternative to SIFT or SURF,” in *2011 International Conference on Computer Vision*, (Barcelona, Spain: IEEE), 2564–2571.

[B48] Shboul Z. A. Alam M. Vidyaratne L. Pei L. Elbakary M. I. Iftekharuddin K. M. (2019). Feature-guided deep radiomics for glioblastoma patient survival prediction. *Front. Neurosci.* 13:966. 10.3389/fnins.2019.00966 31619949 PMC6763591

[B49] Simonyan K. Zisserman A. (2015). “Very deep convolutional networks for large-scale image recognition,” in *The International Conference on Learning Representations*, (La Jolla, CA: ICLR).

[B50] Singla S. Sharma R. (2014). Medical image stitching using hybrid of sift & surf techniques. *Int. J. Adv. Res. Electron. Commun. Eng.* 3 838–842.

[B51] Song Q. Guo X. Zhang L. Yang L. Lu X. (2021). New approaches in the classification and prognosis of sign clusters on pulmonary CT images in patients with multidrug-resistant tuberculosis. *Front. Microbiol.* 12:714617. 10.3389/fmicb.2021.714617 34671326 PMC8521176

[B52] Su H. Lin B. Huang X. Li J. Jiang K. Duan X. (2021). MBFFNet: multi-branch feature fusion network for colonoscopy. *Front. Bioeng. Biotechnol.* 9:696251. 10.3389/fbioe.2021.696251 34336808 PMC8317500

[B53] Suzuki K. Yan P. Wang F. Shen D. (2012). Machine learning in medical imaging. *Int. J. Biomed. Imaging* 2012:123727. 10.1155/2012/123727 22481902 PMC3303553

[B54] Swarnambiga A. Varghese A. Mahendra K. Ganapathy K. (2019). “Medical image retrieval using Resnet-18,” in *Medical Imaging 2019: imaging Informatics for Healthcare, Research, and Applications: international Society for Optics and Photonics*, (New York: ACM), 1095410.

[B55] Tian Q. Yan L.-F. Zhang X. Zhang X. Hu Y.-C. Han Y. (2018). Radiomics strategy for glioma grading using texture features from multiparametric MRI. *J. Magn. Reson. Imaging* 48 1518–1528. 10.1002/jmri.26010 29573085

[B56] Vanino E. Tadolini M. Evangelisti G. Zamparini E. Attard L. Scolz K. (2020). Spinal tuberculosis: proposed spinal infection multidisciplinary management project (SIMP) flow chart revision. *Eur. Rev. Med. Pharmacol. Sci.* 24 1428–1434. 10.26355/eurrev_202002_20201 32096192

[B57] Wang L. Chang C. Liu Z. Huang J. Liu C. Liu C. (2021). A Medical Image Fusion Method Based on SIFT and Deep Convolutional Neural Network in the SIST Domain. *J. Healthc. Eng.* 2021:9958017. 10.1155/2021/9958017 PMC8081630 33968357

[B58] Wang S. Burtt K. Turkbey B. Choyke P. Summers R. M. (2014). Computer aided-diagnosis of prostate cancer on multiparametric MRI: a technical review of current research. *Biomed Res. Int.* 2014:789561. 10.1155/2014/789561 25525604 PMC4267002

[B59] Wang S.-H. Govindaraj V. V. Górriz J. M. Zhang X. Zhang Y.-D. (2021). Covid-19 classification by FGCNet with deep feature fusion from graph convolutional network and convolutional neural network. *Inf. Fusion* 67 208–229. 10.1016/j.inffus.2020.10.004 33052196 PMC7544601

[B60] Wang W. Shi L. Yin A. Mao Z. Maitland E. Nicholas S. (2015). Primary care quality among different health care structures in Tibet, China. *Biomed Res. Int.* 2015:206709. 10.1155/2015/206709 25861619 PMC4377353

[B61] Win K. P. Kitjaidure Y. (2018). “Biomedical images stitching using ORB feature based approach,” in *2018 International Conference on Intelligent Informatics and Biomedical Sciences (ICIIBMS)*, (Bangkok, Thailand: IEEE), 221–225.

[B62] Xie Y. Zhang J. Xia Y. Fulham M. Zhang Y. (2018). Fusing texture, shape and deep model-learned information at decision level for automated classification of lung nodules on chest CT. *Inf. Fusion* 42 102–110. 10.1016/j.inffus.2017.10.005

[B63] Yang Y. Yan L.-F. Zhang X. Han Y. Nan H.-Y. Hu Y.-C. (2018). Glioma grading on conventional MR images: a deep learning study with transfer learning. *Front. Neurosci.* 12:804. 10.3389/fnins.2018.00804 30498429 PMC6250094

[B64] Zhang H. Xu H. Tian X. Jiang J. Ma J. (2021). Image fusion meets deep learning: a survey and perspective. *Inf. Fusion* 76 323–336. 10.1016/j.inffus.2021.06.008

[B65] Zhang N. Zeng X. He L. Liu Z. Liu J. Zhang Z. (2019). The value of MR imaging in comparative analysis of spinal infection in adults: pyogenic versus tuberculous. *World Neurosurg.* 128 806–813. 10.1016/j.wneu.2019.04.260 31103765

[B66] Zhu S. Xia L. Yu S. Chen S. Zhang J. (2017). The burden and challenges of tuberculosis in China: findings from the Global Burden of Disease Study 2015. *Sci. Rep.* 7:14601. 10.1038/s41598-017-15024-1 29097809 PMC5668247

